# Oral microbiota and serum metabolomics features in newly diagnosed anti-AchR antibody-positive myasthenia gravis patients

**DOI:** 10.3389/fimmu.2026.1837031

**Published:** 2026-09-10

**Authors:** Chao Huang, Zhihui Duan, Yan Li, RuiHua Liu, Dandan Shang, Yunting Zhang, Yi Liu, Yuao Wang, Xue Zhao, Yunke Zhang, Feng Gao

**Affiliations:** 1 Department of Encephalopathy, The First Affiliated Hospital of Henan University of Chinese Medicine, Zhengzhou, China; 2 Department of Neurology, Luoyang Central Hospital Affiliated to Zhengzhou University, Luoyang Cerebrovascular Disease (Stroke) Clinical Medical Research Center, Regional Medical Center for Neurological Diseases of Henan Province, Luoyang Key Laboratory of Multi-omics Research and Application in Neurological Diseases, Luoyang, China; 3 Department of Neurology, Henan Medical University, Xinxiang, China; 4 Department of Neuroimmunology, Henan Institute of Medical and Pharmaceutical Sciences, Zhengzhou University, Zhengzhou, China; 5 The First Clinical Medical College, Henan University of Chinese Medicine, Zhengzhou, China

**Keywords:** 16S rRNA, biomarker, metabolomics, myasthenia gravis, oral microbiota

## Abstract

**Background:**

Myasthenia gravis (MG) is an autoimmune disorder mediated by autoantibodies. While research regarding the microbiota-gut-brain axis has expanded significantly in recent years, the role of the oral microbiota in MG and its potential association with systemic metabolism remain poorly understood. To address this, we conducted a case-control study to investigate potential alterations in the oral microbiota and serum metabolites in newly diagnosed and treatment-naïve patients with MG.

**Methods:**

In total, 38 newly diagnosed and treatment-naïve anti-AchR antibody-positive MG patients were recruited, including 19 with ocular myasthenia gravis (OMG) and 19 with generalized myasthenia gravis (GMG), along with 29 healthy controls (HCs). Saliva and serum samples were collected for 16S rRNA gene high-throughput sequencing and untargeted metabolomic profiling, respectively. Data processing and integration were performed using multiple bioinformatics and statistical methods, including α- and β-diversity analyses, LEfSe, Spearman correlation, and metabolic pathway enrichment analysis.

**Results:**

Compared with HCs, patients with OMG and GMG exhibited significant dysbiosis. At the phylum level, the abundance of Bacillota was significantly increased, whereas that of Bacteroidota and Campylobacterota was significantly decreased. At the genus level, the abundances of *Streptococcus* and *Bifidobacterium* were significantly elevated; in contrast, those of *Prevotella* and others were significantly reduced. Serum metabolomics analysis identified distinct metabolic disorders in MG patients, involving key pathways such as lipid metabolism, neuroactive ligand-receptor interaction, and amino acid metabolism. Correlation network analysis further revealed significant associations between specific oral genera (*Streptococcus*, *Bifidobacterium*) and differential metabolites primarily involved in lipid metabolism, amino acid metabolism, and terpenoid and polyketide metabolism in both OMG and GMG populations.

**Conclusions:**

In MG patients, significant oral dysbiosis may be associated with MG-related metabolic alterations by disrupting metabolic pathways, such as those associated with lipid and amino acid metabolism.

## 1 Introduction

Myasthenia gravis (MG) is a chronic, autoantibody-mediated autoimmune disease primarily characterized by impaired transmission at the neuromuscular junction, leading to fluctuating muscle weakness and fatigability in skeletal muscles. The pathophysiological basis of this disease mainly involves an immune attack mediated by autoantibodies targeting the acetylcholine receptor (AChR) or other muscle-specific antigens, such as muscle-specific tyrosine kinase (MuSK) and low-density lipoprotein receptor-related protein 4 (LRP4) (1). Although the antibody-mediated pathogenesis of MG has been well established, clinical manifestations and disease severity vary substantially among patients. This heterogeneity cannot be fully explained by autoantibody status alone, suggesting that additional environmental, immune, and metabolic factors may be associated with disease development and progression.

In recent years, the complex relationship between microbial dysbiosis and autoimmune diseases has received increasing attention. The gut microbiota maintains host immune homeostasis and regulates the differentiation and function of immune cells through the production of metabolites and the modulation of diverse signaling pathways (2). Studies have shown that gut microbiota composition in MG patients differs significantly from that of healthy individuals, characterized by reduced alpha-diversity and alterations in the abundance of specific bacterial genera. These alterations may influence systemic immune responses through changes in microbial metabolites and immune-regulatory pathways (3, 4). Furthermore, serum metabolites, as end products of biochemical reactions, dynamically reflect the systemic metabolic status of patients in real time. Accordingly, studies have increasingly focused on changes in the serum metabolomic profile of MG patients to identify biomarkers related to disease diagnosis, classification, and prognosis (5). However, although gut microbiota and circulating metabolites are closely interconnected (6), the precise mechanisms by which microbial and metabolic disturbances interact to promote immune dysregulation in MG remain incompletely understood.

The oral cavity, constituting the initial segment of the digestive tract, harbors a complex microbial community second only to the gut in taxonomic diversity and total microbial load, and represents a primary source of the gut microbiota (7). Compared to gut samples, saliva offers unique advantages, such as non-invasive collection and ease of storage, making it a convenient resource for the discovery of novel disease biomarkers. Our previous study demonstrated that the oral microbiota was significantly altered in anti-AChR antibody-positive MG patients, characterized by changes in microbial community structure and differential abundances of several bacterial genera, including increased Streptococcus, Rothia, and Lachnoanaerobaculum, and decreased Neisseria, Haemophilus, and Treponema (8). Nevertheless, current knowledge remains limited. Previous studies have mainly focused on describing compositional differences in the oral microbiota between MG patients and healthy controls, whereas the relationship between salivary microbial alterations and clinical features of MG, such as disease severity and clinical subtype, has not been systematically clarified. Moreover, whether salivary microbiota alterations are associated with systemic metabolic disturbances in MG remains largely unknown.

Therefore, in the present study, we characterized the salivary microbiome and serum metabolomic profiles of patients with MG and explored their associations with disease status and clinical subtype. By integrating salivary microbial features, circulating metabolites, and clinical parameters, this study aimed to clarify potential microbiota–metabolite interactions involved in MG and to identify candidate biomarkers related to disease assessment. These findings may provide new insights into the microbial and metabolic factors contributing to MG heterogeneity and offer a scientific basis for early diagnosis, severity evaluation, and personalized management of this disease.

## 2 Materials and methods

### 2.1 Participants

This study was approved by the Ethics Committee of Luoyang Central Hospital (Approval No. 2023-YX-011-12). All participants provided written informed consent before enrollment. The case group consisted of 38 anti-AChR antibody-positive MG patients diagnosed at the Luoyang Central Hospital Affiliated to Zhengzhou University, and Henan Institute of Medical and Pharmaceutical Sciences between April 2023 and May 2024. All patients met the Chinese Guidelines for the Diagnosis and Treatment of Myasthenia Gravis (2020 edition), and diagnoses and classifications were confirmed independently by two neurologists. Patients were clinically classified into an ocular myasthenia gravis (OMG) group (*n* = 19) and a generalized myasthenia gravis (GMG) group (*n* = 19). Concurrently, 29 healthy volunteers were recruited through public advertisements as the healthy control (HC) group. Frequency matching by age and sex was used between the overall MG case group and the HC group. OMG and GMG were clinical subgroups and were not matched to each other. Eligibility was confirmed via health questionnaires and interviews, which verified the absence of organic diseases, systemic diseases, oral diseases, or a family history of autoimmune diseases. To ensure data consistency, participants were excluded based on the following criteria: use of corticosteroids, immunosuppressants, biologics, antibiotics affecting the oral microbiota, probiotics, or acid-suppressing drugs (e.g., proton pump inhibitors) within the past month, a history of chronic gastrointestinal diseases or gastrointestinal surgery; and pregnancy or lactation. All saliva and serum samples were collected before corticosteroid, immunosuppressive, or other systemic treatments.

### 2.2 Sample collection

A total of 67 participants provided saliva samples, comprising 19 patients with OMG, 19 with GMG, and 29 HCs. Serum metabolomic data were available for 65 participants, comprising 18 patients with OMG, 18 with GMG, and 29 HCs.

For blood collection, participants fasted for 12 hours, and 5 mL of venous blood was drawn in the morning. Saliva samples were collected using specialized collection tubes. Participants were instructed to abstain from eating, drinking, smoking, or chewing gum for 30 minutes prior to sampling. All samples were transported on ice within 1 hour and stored at −80 °C. QMG assessments were performed between 08:00 and 10:00 on the same morning as biospecimen collection. Acetylcholinesterase inhibitors were withheld for at least 12 hours before the assessment, and biological samples were collected before QMG assessment.

### 2.3 16S rRNA sequencing

Total genomic DNA was extracted using the E.Z.N.A. Stool DNA Kit (Omega Bio-tek, Inc., GA, USA) following the manufacturer’s instructions for bacterial DNA extraction. PCR amplification of the V3–V4 region of the bacterial 16S rRNA gene was performed on an EasyCycler 96 PCR system (Analytik Jena Corp., AG) using forward primer 341F (5′-CCTACGGGNGGCWGCAG-3′) and reverse primer 805R (5′-GACTACHVGGGTATCTAATCC-3′). The products from different samples were indexed and mixed at equal ratios for sequencing. Sequencing was performed by Shanghai Mobio Biomedical Technology Co., Ltd (Shanghai, China) using the NextSeq 2000 platform (Illumina Inc., USA) according to the manufacturer’s instructions.

### 2.4 Bioinformatics and statistical analysis

Raw 16S rRNA gene sequencing reads were processed using QIIME 2 (version 2024.2). Quality filtering, denoising, paired-end read merging, and chimera removal were performed using the dada2 denoise-paired pipeline implemented in q2-dada2 (version 2024.2.0). Amplicon sequence variants (ASVs) with a total abundance of fewer than 2 reads or detected in fewer than 2 samples were removed. Sequences classified as mitochondrial, chloroplast, or archaeal were excluded. Samples with fewer than 10,000 reads were excluded before downstream analyses. The remaining ASVs were taxonomically annotated using the SILVA database (SSU138). Alpha diversity was assessed at the ASV level using the ACE, Chao1, Shannon, and Simpson indices. Beta diversity was evaluated by principal coordinates analysis (PCoA) based on Bray–Curtis, unweighted UniFrac, and weighted UniFrac distances. Overall differences in microbial community composition among groups were assessed using PERMANOVA (Adonis method). Microbial composition from the phylum to genus level was visualized using stacked bar plots generated in R. Differences in relative abundance among the three groups were assessed using the Kruskal-Wallis test, followed by Dunn’s *post hoc* test for pairwise comparisons with false discovery rate (FDR) correction. An FDR-adjusted P value <0.05 was considered statistically significant. To assess the robustness of the primary comparisons and potential residual confounding by the frequency-matching factors, CLR-transformed microbial abundances were modeled using limma with group, age, and sex as predictors; P values were adjusted using the Benjamini–Hochberg procedure. These sensitivity analyses used the 65 samples with matched clinical and abundance data and were used to assess robustness of the primary analyses. Linear discriminant analysis effect size (LEfSe) was performed at the genus level to identify differentially abundant genera (LEfSe v1.1; https://github.com/SegataLab/lefse). Microbial functional genes and metabolic pathways were predicted using PICRUSt2 (https://github.com/picrust/picrust2/wiki), and differentially abundant pathways among groups were identified using LEfSe analysis.

Raw sequencing data for the V3-V4 region of the 16S rRNA gene and accompanying information are available in the Sequence Read Archive database under accession number PRJNA1415981.

### 2.5 Metabolic profiling and association analysis

Serum metabolomic profiling was performed using ultra-high-performance liquid chromatography-mass spectrometry (UHPLC-MS). All samples were analyzed in a single analytical batch, and the injection order was randomized before acquisition. Pooled quality-control samples, prepared from aliquots of the study samples, were injected at predefined intervals to monitor analytical stability and instrumental drift. Raw mass-spectrometry data were processed using XCMS for peak detection, filtering, and alignment. Missing values were imputed using the k-nearest-neighbors method, and features with a coefficient of variation >30% in quality-control samples were excluded. Principal component analysis (PCA), partial least squares-discriminant analysis (PLS-DA), and orthogonal partial least squares-discriminant analysis (OPLS-DA) were performed using the R package “Ropls” for dimensionality reduction and multivariate analysis. Models were evaluated using the internal cross-validation procedure implemented in the ropls package and 200 permutation tests. To assess potential residual confounding by the frequency-matching factors, log2-transformed metabolite abundances were modeled using limma with group, age, and sex as predictors, with Benjamini–Hochberg FDR correction. Differential metabolite analysis was conducted for both pairwise and three-group comparisons. Metabolite abundance data were first log2-transformed, followed by the Shapiro-Wilk test for normality and Levene’s test for homogeneity of variance. Appropriate statistical tests were selected according to data distribution and variance homogeneity. For normally distributed data with equal variances, Student’s t-test or one-way ANOVA was used, followed by FDR correction or Tukey’s *post hoc* test, respectively. For normally distributed data with unequal variances, Welch’s t-test or Welch’s ANOVA was used, followed by P-value correction or the Games-Howell *post hoc* test, respectively. For non-normally distributed data, the Mann-Whitney U test or Kruskal-Wallis test was used, followed by FDR correction or Dunn’s *post hoc* test, respectively. In pairwise comparisons, metabolites with an FDR-adjusted P value < 0.05 and a VIP score > 1 in the OPLS-DA model were defined as differentially abundant metabolites. In three-group comparisons, metabolites with an adjusted P value < 0.05 in any pairwise comparison were considered differentially abundant. The selected differentially abundant metabolites were subjected to functional pathway enrichment and topological analyses using MetaboAnalystR. Enriched pathways were visualized using Pathview to explore the relationships between differential metabolites and pathway maps. Spearman correlation analysis was used to assess associations between key differentially abundant bacterial taxa and metabolites. In the figures and tables, statistical significance is denoted as follows: *P < 0.05, **P < 0.01, and ***P < 0.001.

## 3 Results

### 3.1 General characteristics

A total of 67 saliva samples were collected from 38 anti-AChR antibody-positive MG patients, including 19 patients with OMG and 19 with GMG, and from 29 age- and sex-frequency-matched HCs. Serum metabolomic data were available for 65 participants, including 18 patients with OMG, 18 with GMG, and 29 HCs. One participant in each MG subgroup provided saliva but not serum, resulting in one fewer case per subgroup for serum analyses. The clinical characteristics of all participants are summarized in **Table 1**.

**Table 1:** Detailed clinical characteristics of all participants.

Characteristic	OMG	GMG	HC	*P*
Sample size	19	19	29	–
Female, n(%)	11 (57.8%)	8 (42.1%)	16 (55.2%)	0.57
Age, years, mean ± SD	49.8 ± 16.1	56.3 ± 15.7	52.3 ± 14.1	0.737
BMI, mean ± SD	26.5 ± 4.6	24.8 ± 3.0	25.0 ± 4.0	0.429
Time from symptom onset to sampling, weeks, median (range)	4.00 (1.00-50.00)	4.00 (1.00-48.00)	N/A	0.368
Thymoma/thymic hyperplasia, n (%)	2 (10.5%)	3 (15.8%)	N/A	1.00
Thymectomy, n (%)	1 (5.3%)	1 (5.3%)	N/A	1.00
QMG score, mean ± SD	3.26 ± 1.15	9.79 ± 4.35	N/A	<0.001

Data are presented as mean ± SD or n (%). *P* values were derived from one-way ANOVA (age), Chi-square test (sex), Kruskal-Wallis test(BMI), Mann‑Whitney U test (time from symptom onset to sampling) and Fisher’s exact test (thymoma, thymectomy). QMG scores between OMG and GMG were compared using Welch’s t-test. The time from symptom onset to sampling was available only for MG patients; therefore, HC group is marked as N/A. The healthy control group had no thymic abnormalities or history of thymectomy by definition. OMG, ocular myasthenia gravis; GMG, generalized myasthenia gravis; HC, healthy control; QMG, Quantitative Myasthenia Gravis; N/A, not applicable.

### 3.2 MG patients display significant structural and compositional oral dysbiosis

**Figure 1:**
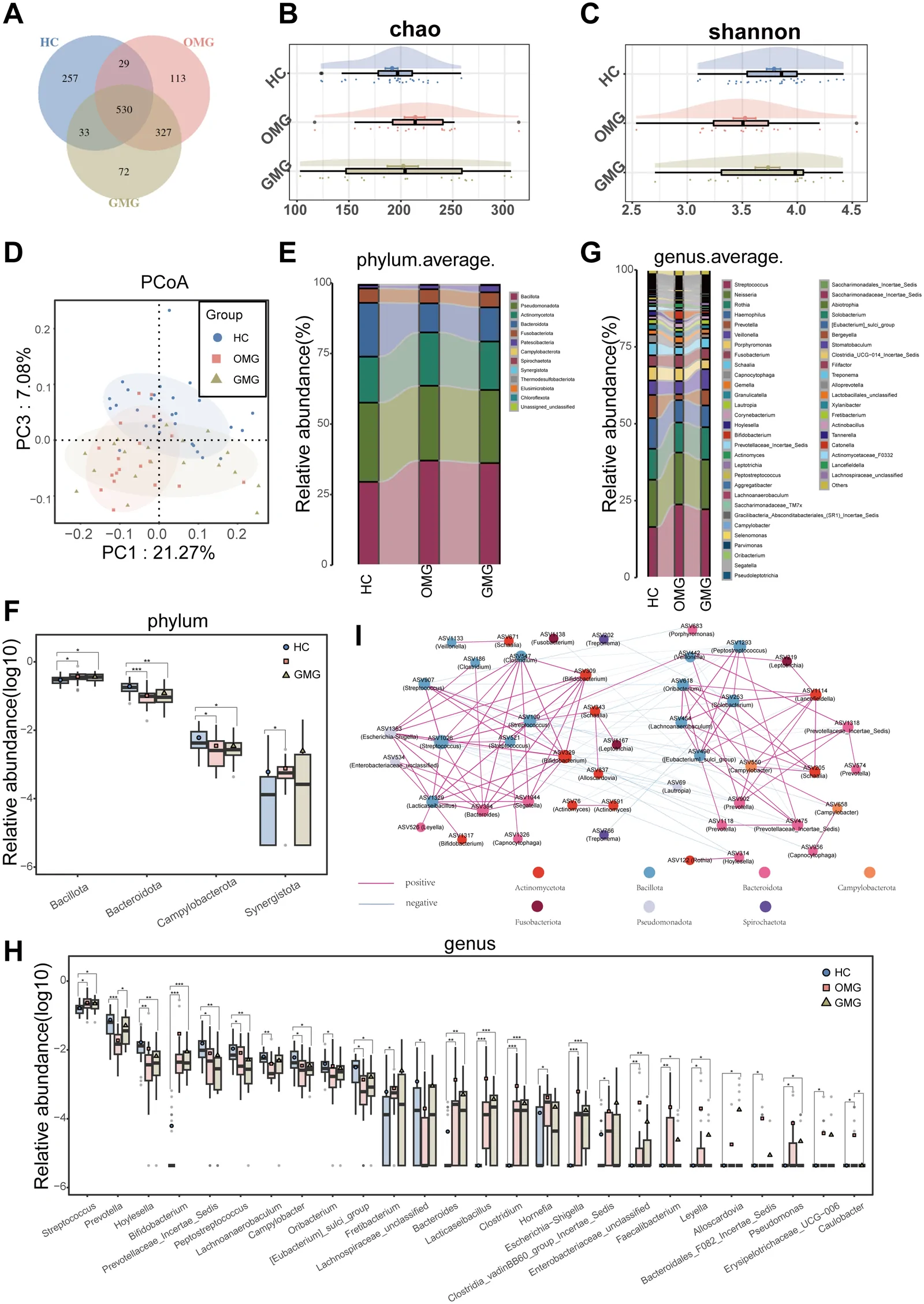
Comparative analysis of oral microbial diversity and composition among the ocular myasthenia gravis (OMG, *n* = 19), generalized myasthenia gravis (GMG, *n* = 19), and healthy control (HC, *n* = 29) groups. **(A)** Venn diagram based on the amplicon sequence variants (ASV) abundance table. **(B)** Alpha-diversity estimated by the Chao index. **(C)** Alpha-diversity estimated by the Shannon index. **(D)** Beta-diversity based on principal coordinates analysis (PCoA) using the weighted UniFrac algorithm. **(E)** Composition of the microbial community at the phylum level. **(F)** Differential relative abundances at the phylum level. **(G)** Composition of the oral microbial community at the genus level. **(H)** Differential relative abundances at the genus level. **(I)** SparCC co-occurrence network of differentially abundant bacterial taxa identified in intergroup comparisons. Edges represent positive or negative taxon-taxon associations. *P* values for the intergroup differential-abundance comparisons were calculated using the Kruskal-Wallis test. *P < 0.05, **P < 0.01, ***P < 0.001.

To investigate the differences in salivary microbiota between the MG and HC groups, 16S rRNA gene sequencing was performed on all saliva samples. A total of 1,361 ASVs were detected, among which 530 ASVs were shared by the HC, OMG, and GMG groups. In addition, 257, 113, and 72 ASVs were unique to the HC, OMG, and GMG groups, respectively (**Figure 1A**). Alpha-diversity was assessed at the ASV level using the Chao richness index and Shannon diversity index. The Chao richness index and Shannon diversity index did not show robust significant differences among the three groups, although the OMG group tended to show lower Shannon diversity than the HC group (**Supplementary Table 1**; **Figures 1B, C**).

Intergroup β-diversity was further assessed through PCoA. As shown in **Figure 1D**, the HC group exhibited distinct spatial separation from both the OMG and GMG groups, indicating that the overall structure of the oral microbiota differed significantly between MG patients and HCs (HC *vs*. OMG, *P* = 0.001; HC *vs*. GMG, *P* = 0.004). However, no significant difference in oral microbiota structure was found between the OMG and GMG groups (*P* = 0.108). These findings suggested that the overall structure of the oral microbiota in MG patients was significantly distinct from that in healthy individuals, whereas no differences were detected between disease subtypes.

To investigate the differences in microbiota composition among the groups, we analyzed relative bacterial abundances at the phylum and genus levels. Although the core microbial taxa were similar across groups, their relative abundances exhibited significant fluctuations (**Figures 1E, G**). At the phylum level, compared with the HC group, the relative abundance of Bacillota was significantly elevated in both the OMG and GMG groups, whereas those of Bacteroidota and Campylobacterota were markedly decreased (**Figure 1F**). At the genus level, the relative abundances of *Streptococcus* and *Bifidobacterium* were significantly higher in the OMG and GMG groups than in the HC group. In contrast, the abundances of *Prevotella*, *Hoylesella*, Prevotellaceae_Incertae_Sedis, *Peptostreptococcus*, and *Campylobacter*, among others, were significantly reduced (**Figure 1H**). Sensitivity analyses for the microbiome data showed that, across overall and pairwise group comparisons, most taxa significant in the primary analysis (FDR < 0.05) remained significant after covariate adjustment (P < 0.05), and the majority also retained FDR < 0.05. The main results at phylum and genus levels were generally robust (**Supplementary Table 18**).

To examine co-occurrence patterns among differentially abundant ASVs, we constructed a SparCC co-occurrence network to visualize positive and negative taxon–taxon associations (**Figure 1I**). This network describes co-occurrence relationships among the selected taxa and was not used to infer associations between individual taxa and MG disease status. The higher relative abundances of *Streptococcus* and *Bifidobacterium* in the MG groups than in HCs were identified in the intergroup abundance analyses described above (**Figure 1H**).

Collectively, these findings indicate that MG was associated primarily with alterations in oral microbial community structure and taxonomic composition, without a consistent reduction in alpha diversity after multiple-testing correction. These findings suggest that this oral microecological imbalance may be associated with MG pathophysiology. However, further analyses are warranted to explore and identify the differences between OMG and GMG.

### 3.3 Oral microbiota functions differ among the OMG, GMG, and HC groups

**Figure 2:**
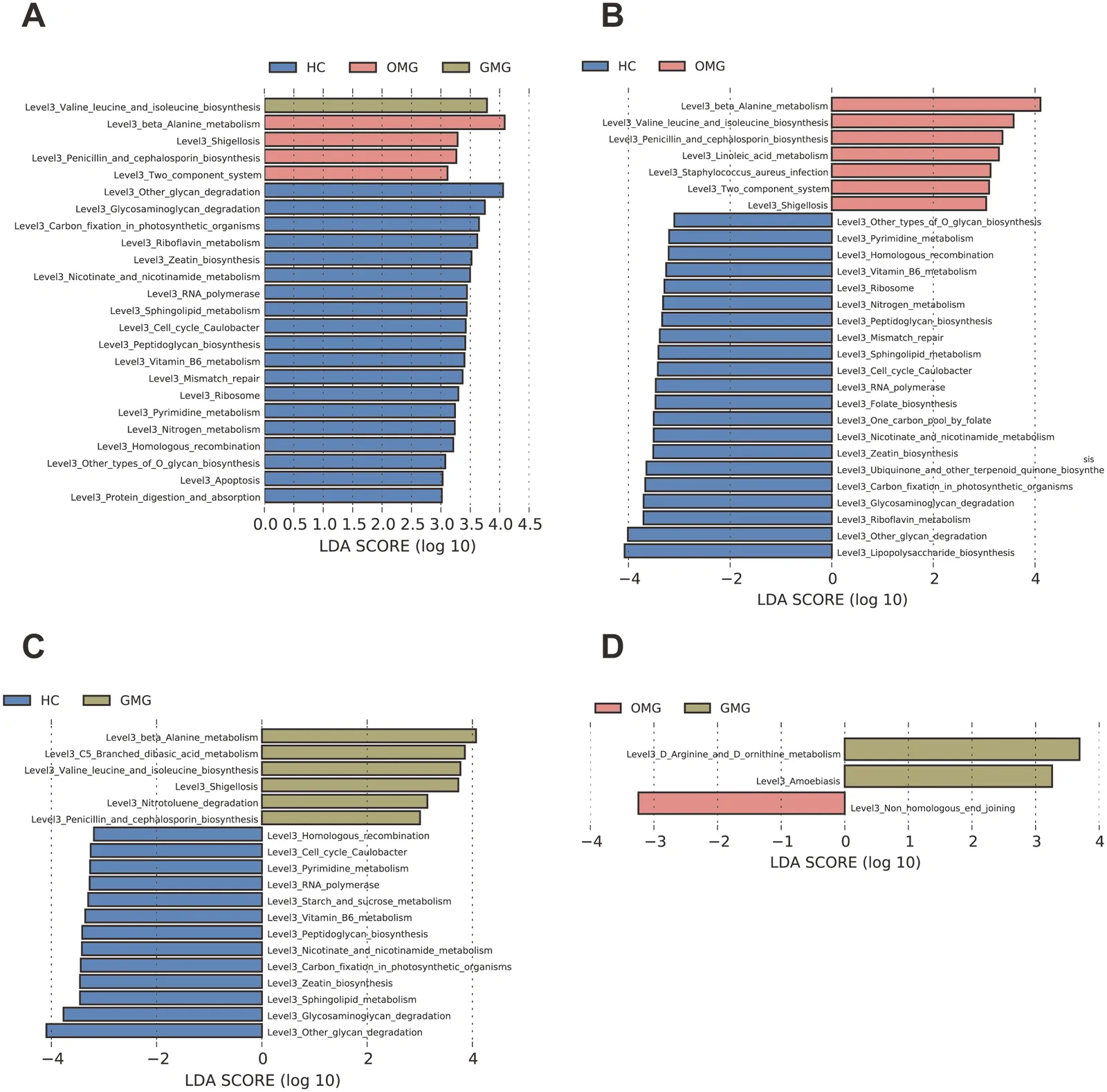
Functional prediction of differential oral microbiota among the ocular myasthenia gravis (OMG, n = 19), generalized myasthenia gravis (GMG, n = 19), and healthy control (HC, n = 29) groups. Functional prediction of the 16S sequencing data was performed based on the KEGG Pathway database. LEfSe analysis was subsequently conducted to identify significantly differential metabolic pathways (Level 3) among the OMG(red), GMG(green), and HC(blue) groups. **(A)** Overall comparison among the HC, OMG, and GMG groups; **(B)** Pairwise comparison between HC (negative scores) and OMG (positive scores); **(C)** Pairwise comparison between HC (negative scores) and GMG (positive scores); **(D)** Pairwise comparison between OMG (negative scores) and GMG (positive scores).

Using PICRUSt functional prediction coupled with LEfSe analysis (LDA score ≥ 3, *P* < 0.05), we compared the differences in metabolic functions for the oral microbiota showing differential abundance among the OMG, GMG, and HC groups based on KEGG pathways. Multiple pathways were enriched across groups (**Figure 2A**). The oral microbiota exhibiting differential abundance between the HC group and both the OMG and GMG groups were significantly associated with beta-alanine metabolism; valine, leucine and isoleucine biosynthesis; penicillin and cephalosporin biosynthesis; and shigellosis (**Figures 2B, C**). Further comparison of the differentially abundant microbiota between the OMG and GMG groups (**Figure 2D**) identified differences in pathways such as D-arginine and D-ornithine metabolism, amoebiasis, and non-homologous end joining. Collectively, these results demonstrated that the predicted functional profiles of the oral microbiota in both OMG and GMG patients are significantly distinct from those of healthy individuals. Functional divergence was also observed in several metabolic and repair pathways between the two patient populations, suggesting that their microbial communities may contribute to their distinct clinical classifications.

### 3.4 MG patients present marked serum metabolic profile disorders, and OMG and GMG show unique metabolic features

**Figure 3:**
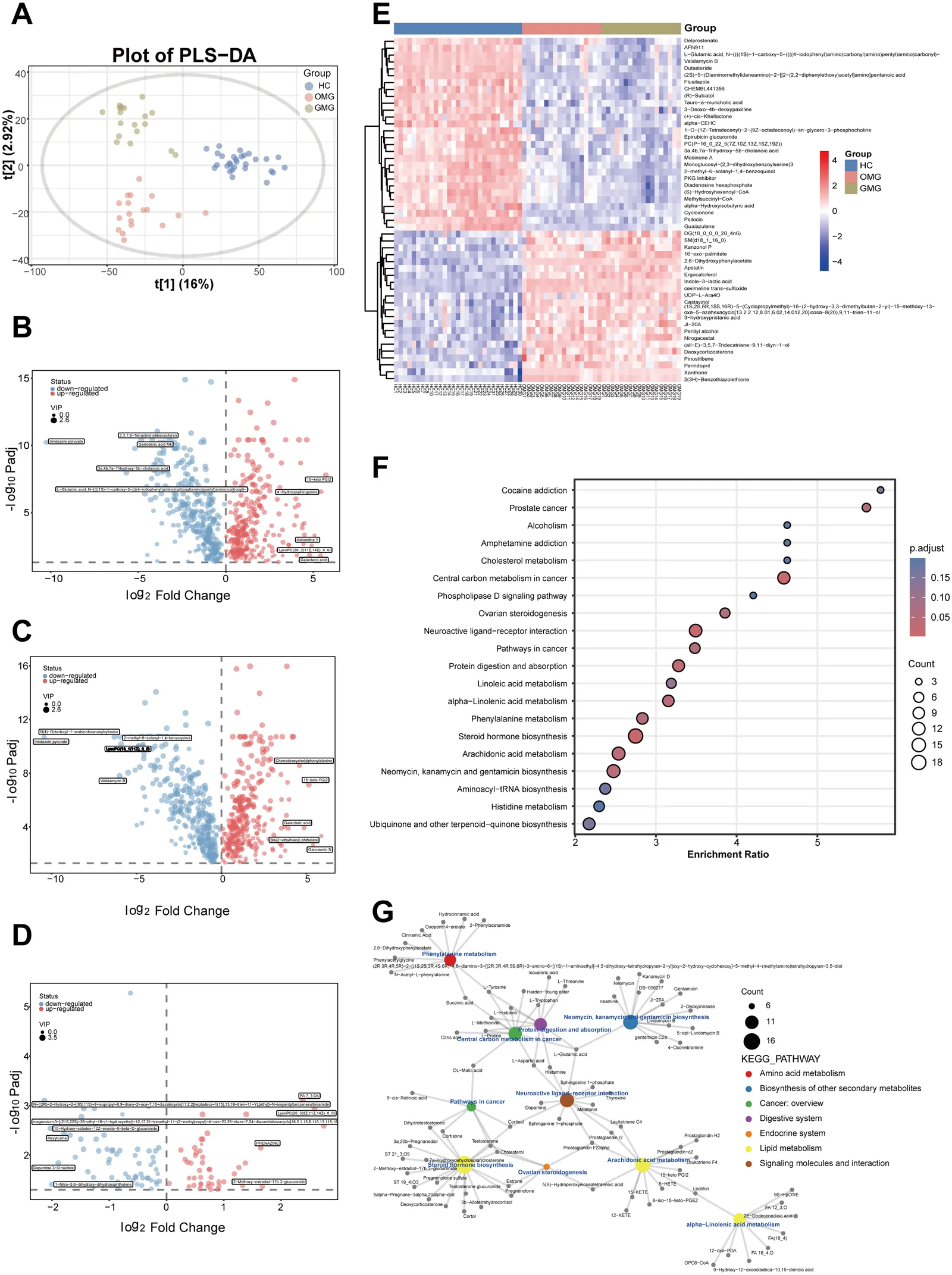
Serum metabolome analysis among the ocular myasthenia gravis (OMG, *n* = 18), generalized myasthenia gravis (GMG, *n* = 18), and healthy control (HC, *n* = 29) groups. **(A)** PLS-DA score plot showing the distribution trends of metabolites across the three groups. **(B)** Distribution and variation trends of differential metabolites in the OMG group compared to the HC group. **(C)** Distribution and variation trends of differential metabolites in the GMG group compared to the HC group. **(D)** Distribution and variation trends of differential metabolites in the OMG group compared to the GMG group. **(E–G)** Heatmap of the differential metabolites among the three groups, bubble chart of the KEGG pathway enrichment analysis, and the corresponding network diagram.

To investigate the metabolic characteristics of MG patients, we performed an untargeted metabolomic analysis on serum samples using UHPLC-MS. The generated PLS-DA model revealed a distinct separation in metabolic profiles when comparing the MG cohorts (OMG and GMG) with the HCs, indicating that the pathological state of MG is associated with significant metabolic disturbances (**Supplementary Tables 8–10**; **Figure 3A**). Intergroup comparisons revealed a large number of differential metabolites (**Figure 3E**). Intergroup comparisons revealed a large number of differentially abundant metabolites (**Supplementary Tables 11**–**15**; **Figures 3B–D**). Compared with the HC group, 650 metabolites were identified as differentially abundant in the OMG group, and 662 metabolites were identified as differentially abundant in the GMG group. A direct comparison between the OMG and GMG groups identified 113 differentially abundant metabolites, indicating that the two MG subtypes displayed distinct metabolic characteristics. Among these differential metabolites, several lipid-related molecules and amino acid-related metabolites showed prominent intergroup differences. For instance, LysoPC(20:2) and galactaric acid were among the representative metabolites altered in the OMG-related comparison, whereas LysoPC(18:1), imidazole pyruvate, and other metabolites showed marked changes in the GMG-related comparison. The heatmap of the top 50 differentially abundant metabolites further showed that HC, OMG, and GMG samples exhibited distinct metabolic distribution patterns (**Figure 3E**). Sensitivity analyses for the metabolomics data showed that, in the overall comparison and in comparisons with HC, over 90% of metabolites that were significant in the primary analysis (FDR < 0.05) retained P < 0.05 after covariate adjustment, and approximately 85% retained FDR < 0.05. In the GMG vs OMG comparison, most of the originally significant metabolites remained significant after adjustment, showing good consistency. Overall, the main metabolomic findings remained robust after adjusting for covariates (**Supplementary Tables 16, 17**).

KEGG pathway enrichment analysis further revealed that the differentially abundant metabolites were mainly enriched in lipid metabolism, amino acid metabolism, neuroactive ligand–receptor interaction, steroid hormone biosynthesis, and cancer-related metabolic pathways (**Figure 3F**). The metabolite-pathway network further indicated that these altered metabolites were connected with pathways such as arachidonic acid metabolism, α-linolenic acid metabolism, steroid hormone biosynthesis, phenylalanine metabolism, protein digestion and absorption, and neuroactive ligand–receptor interaction (**Figure 3G**).

Collectively, these data demonstrated that patients with MG exhibit significant disturbances in serum metabolic profiles. These not only distinguish healthy individuals from MG patients, but also serve to differentiate between the OMG and GMG subtypes. These metabolic alterations were mainly associated with lipid-mediated inflammatory pathways, amino acid metabolism, and neuroactive signaling-related pathways, suggesting a potential link between systemic metabolic remodeling and MG-related clinical heterogeneity.

### 3.5 *Streptococcus* and *Bifidobacterium* may drive the development of MG via the modulation of host lipid, amino acid, terpenoid, and polyketide metabolism

**Figure 4:**
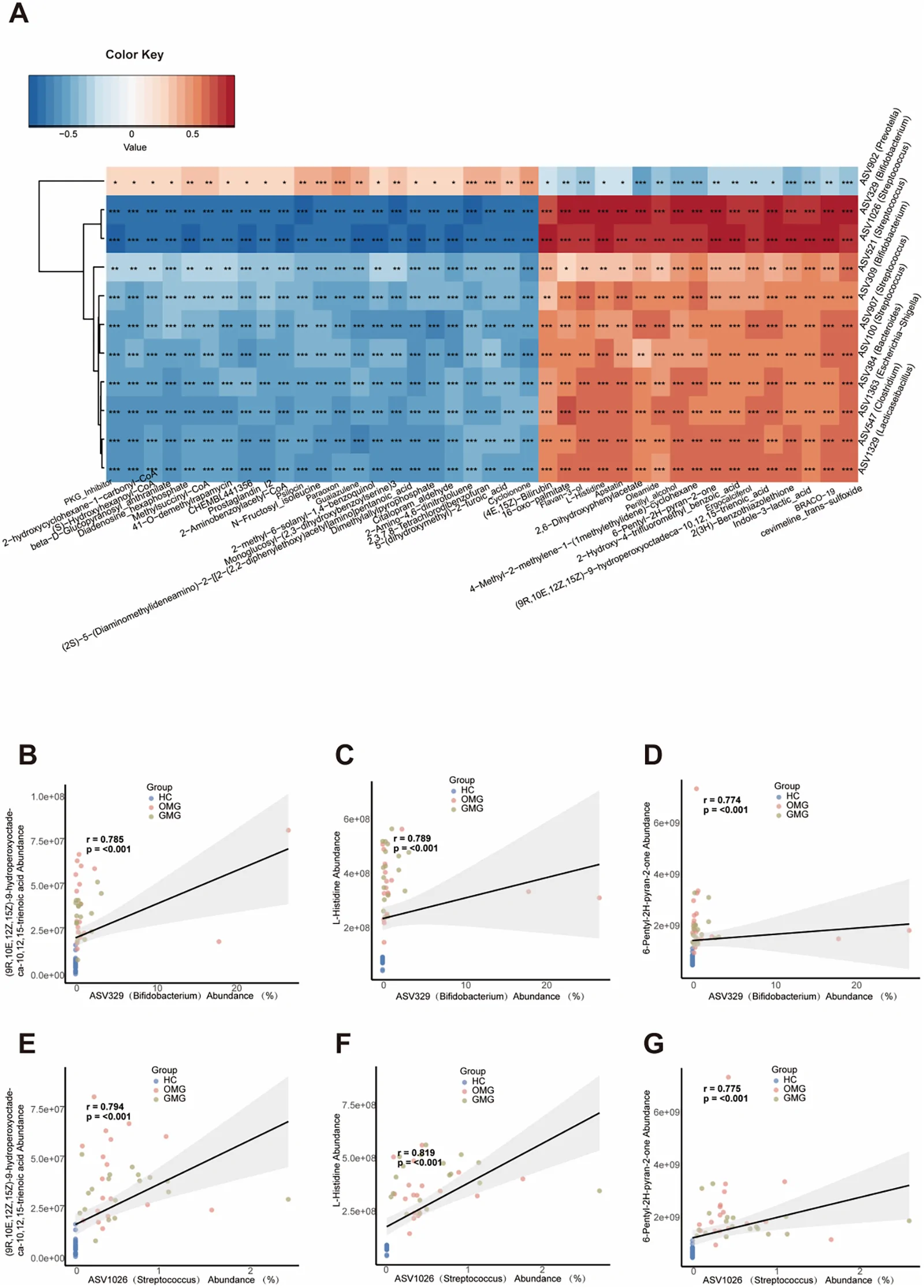
Correlation analysis between 11 differential microbial taxa and differential metabolites among the ocular myasthenia gravis (OMG, *n* = 18), generalized myasthenia gravis (GMG, *n* = 18), and healthy control (HC, *n* = 29) groups. **(A)** Heatmap of Spearman correlation coefficients. **(B–G)** Visualization of selected correlations.

To explore the potential crosstalk between the oral microbiota and host systemic metabolism, we undertook a correlation analysis between differential oral microbiota and differential serum metabolites. The results demonstrated that several MG-associated oral bacteria exhibited significant correlations with a panel of serum metabolites (**Figures 4A–G**). Among these, *Streptococcus* and *Bifidobacterium* showed strong associations with a wide range of metabolites, including bile pigment molecules ((4E,15Z)-bilirubin), lipid metabolism-related molecules (16-oxo-palmitate, oleamide, (9R,10E,12Z,15Z)-9-hydroperoxyoctadeca-10,12,15-trienoic acid), amino acids and their derivatives (L-histidine, indole-3-lactic acid, 2,6-dihydroxyphenylacetate), terpenoids and ketone compounds (perillyl alcohol, 6-pentyl-2H-pyran-2-one, ergocalciferol), and other functional molecules (flavan-3-ol, apstatin, BRACO-19, etc.). These findings suggested that oral *Streptococcus* and *Bifidobacterium* may be associated with MG-related metabolic alterations involving lipid metabolism, amino acid metabolism, and terpenoid and polyketide metabolism.

## 4 Discussion

The oral microbiota comprises the second largest microbial ecosystem in the human body, playing a crucial role in maintaining health through interactions with the host immune system (9). Accumulating evidence has indicated that oral dysbiosis is closely associated with the pathogenesis and progression of multiple autoimmune diseases (10, 11). This exploratory study provides preliminary evidence of substantial oral microbiota disturbances and related systemic metabolic alterations in patients with anti-AChR antibody-positive MG. The integration of 16S rRNA gene sequencing and serum metabolomics allowed for a comprehensive characterization of both OMG and GMG populations.

A primary finding of this study was that oral microbiota dysbiosis in patients with MG was mainly characterized by altered community structure and shifts in the relative abundance of specific taxa, rather than a consistent reduction in overall microbial diversity. Although no robust significant difference in alpha diversity remained after multiple-testing correction, the OMG group showed a tendency toward lower Shannon diversity than the HC group. In contrast, both OMG and GMG patients displayed significant differences in β-diversity relative to the HC group. These findings indicate that the overall richness and diversity of the oral microbiota were not consistently altered in the MG state, whereas the community composition underwent distinct structural changes.

These findings diverge from existing understanding of the gut microbiota (12, 13), suggesting that the disease exerts differential effects on the microecology of distinct niches. Importantly, although OMG and GMG did not exhibit complete separation in overall β-diversity, significant differences were detected in their specific genus-level composition. For instance, the relative abundances of *Prevotella* and *Leptotrichia* were significantly higher in the GMG group than in the OMG group. These specific alterations at the genus level suggest that particular members of the oral microbiota, rather than drastic shifts in the entire community, may be associated with clinical subtyping of MG.

At the phylum level, both OMG and GMG patients consistently exhibited an increase in the abundance of Firmicutes and a decrease in that of Bacteroidetes. This trend is consistent with our previous study on the oral microbiota (8), but contradicts the findings of most investigations on the gut microbiota in MG (14–16). Such discrepancies highlight the unique characteristics of the oral cavity, comprising an independent microecological niche in the context of MG. At the genus level, the consistent increase in *Streptococcus* and *Bifidobacterium* abundance in the oral cavity of MG patients is particularly notable. *Streptococcus* may influence immune regulation through pathways such as PPAR-γ-related signaling, thereby contributing to immune dysregulation (17). Although an increase in *Bifidobacterium* is generally regarded as beneficial, its effects are strain-specific, and this bacterium may also promote inflammation under certain conditions (18). The association between *Bifidobacterium* and MG observed in this study may result from compensatory regulation under disease conditions or the proliferation of specific strains with different functional properties.

To further explore the functional consequences of microbiota alterations, we performed functional prediction using PICRUSt2 and identified differential functional pathways between the two groups via LEfSe analysis. Taxon-inferred functional prediction suggested that oral microbial alterations may be associated with pathways related to amino acid metabolism and the biosynthesis of secondary metabolites. Further metabolomic analysis revealed the presence of extensive metabolic disturbances in both OMG and GMG patients, particularly concentrated in metabolic pathways such as neuroactive ligand-receptor interaction and arachidonic acid metabolism. Studies have demonstrated that pro-inflammatory cytokines, such as TNF-α, IL-1β, and IFN-γ, may influence neuromuscular transmission and immune responses, although they are not classical neuroactive ligands (19–21). Arachidonic acid metabolism generates potent lipid inflammatory mediators, including prostaglandin E2 (PGE2) and leukotrienes (LTs) (22). PGE2 promotes the differentiation of T cells into pro-inflammatory Th17 cells and inhibits Treg function, thereby disrupting immune tolerance and exacerbating autoantibody production (23). LTB_4_, a representative LT product, recruits neutrophils, monocytes/macrophages, and memory T cells to inflammatory sites via BLT1/BLT2 receptors and stimulates these immune cells to release pro-inflammatory factors, including IL-6, TNF-α, and IL-1β (24, 25). These metabolites may be involved in immune and inflammatory processes relevant to MG through multidimensional interactions involving immune regulation, inflammatory responses, and neural signal transmission. Notably, GMG patients exhibited a more complex and specific pattern of metabolite alterations in these pathways, potentially corresponding to more extensive clinical manifestations. Subsequent correlation analysis showed that Streptococcus and Bifidobacterium were significantly associated with differential serum metabolites involved in lipid and amino acid metabolism. This establishes a plausible hypothesis, whereby oral dysbiosis in MG patients participates in immunopathological processes by modulating systemic metabolic homeostasis, specifically through the influence of *Streptococcus* and *Bifidobacterium* on lipid and amino acid metabolic networks. This speculation is consistent with the study of Ding et al., who reported disturbances in amino acid metabolism in the gut microbiota in MG (4, 12), suggesting that the “microbiota-metabolism-immunity” axis may represent a common core mechanism underlying MG pathogenesis, regardless of the ecological niche.

In conclusion, our study observed that patients with MG exhibit oral dysbiosis and serum metabolic profile alterations, some of which are disease-subtype specific. These findings suggest that oral microbiota alterations may be linked to MG-related metabolic changes, particularly those involving lipid and amino acid metabolism. However, this study has several limitations. First, the sample size of each group was relatively small, and no additional neurological disease control group was included; therefore, the MG‑specificity of the observed oral microbial alterations cannot be fully determined. Second, the cross‑sectional design limited our ability to evaluate the dynamic changes in oral microbiota and serum metabolites during disease progression or after treatment. Third, the present study mainly relied on group‑based comparisons to identify differential features, and we have not yet validated the linear relationships between these features and disease severity (e.g., QMG scores) at the continuous variable level. Fourth, potential confounding factors, including diet, oral hygiene, smoking, and alcohol consumption, were not fully standardized or included in multivariable correction. In addition, synchronous fecal samples were lacking, precluding further clarification of the role of the oral-gut microbiota axis in MG.

Future research will address several of these limitations. We intend to conduct prospective longitudinal studies with continuous sampling at early disease onset, at the time of diagnosis, post‑treatment, and during follow‑up to capture the dynamic trajectories of oral microbiota and serum metabolites. Furthermore, we will validate the generalizability of our findings in larger independent cohorts and perform targeted analyses to examine the dose‑response relationships between candidate biomarkers and quantitative measures of disease severity, such as QMG scores. In parallel, animal models and/or *in vitro* experiments will be employed to further explore the specific molecular mechanisms underlying the immunoregulatory effects of key oral bacterial species and their metabolites in MG. These efforts are expected to provide potential targets for the development of novel therapeutic intervention strategies for this disease.

## Data Availability

The datasets presented in this study can be found in online repositories. The names of the repository/repositories and accession number(s) can be found in the article/**Supplementary Material**.
